# Prevention of Arthritis by Locally Synthesized Recombinant Antibody Neutralizing Complement Component C5

**DOI:** 10.1371/journal.pone.0058696

**Published:** 2013-03-07

**Authors:** Paolo Durigutto, Paolo Macor, Federica Ziller, Luca De Maso, Fabio Fischetti, Roberto Marzari, Daniele Sblattero, Francesco Tedesco

**Affiliations:** 1 Department of Life Sciences, University of Trieste, Trieste, Italy; 2 Dipartimento Universitario Clinico di Scienze Mediche, Chirurgiche e della Salute, University of Trieste, Trieste, Italy; 3 Department of Medical Sciences and IRCAD, University of Eastern Piedmont, Novara, Italy; University of Leicester, United Kingdom

## Abstract

Treatment of patients suffering from chronic diseases such as rheumatoid arthritis with recombinant antibodies is time consuming and fairly expensive and can be associated with side effects due to generalized depletion of the target molecule. We have addressed these issues by developing an alternative approach consisting of the intraarticular injection of a DNA vector encoding for the anti-C5 neutralizing recombinant miniantibody MB12/22. This method allows local production of the antibody in sufficient amount to be effective in preventing joint inflammation in a rat model of antigen-induced arthritis. Injection of the DNA vector in a right knee of normal rats resulted in the production of the minibody detected in the synovial washes by western blot with a strong signal peaking at 3 days after administration. DNA encoding for the minibody was shown for 14 days in the synovial tissue and was undetectable in the controlateral knee and in other organs. The preventive effect of this approach was evaluated in rats receiving a single injection of the vector 3 days before the induction of antigen-induced arthritis and analyzed 3 days later. The treated rats exhibited a lower increase in swelling, associated with a lower number of PMN in the articular washes and reduced deposition of C9 in synovial tissue compared to control rats. These results suggest that treating the inflamed joints with a vector that induces a local production of a neutralizing anti-C5 antibody may represent a useful strategy to inhibit in situ complement activation and to treat patients with monoarthritis. Moreover, this approach may be adopted as a novel therapeutic strategy to prevent monoarthritis as an alternative to local treatment with antibodies commonly used in this form of arthritis, with the advantages of the lower cost and the longer persistence of antibody production.

## Introduction

Rheumatoid arthritis (RA) is an autoimmune disease characterized by chronic inflammation of synovial tissue infiltrated by T, B, dendritic cells and macrophages producing pro-inflammatory cytokines and auto-antibodies, that lead to cartilage destruction, bone erosion and eventually to joint deformities and disability [1].

In recent years, the development of biologic agents that target specific soluble or membrane-bound molecules has revolutionized the treatment of RA in patients with inadequate response to disease-modifying antirheumatic drugs (DMARDs). Tumor necrosis factor alpha (TNF-alpha) is an ideal target of these biologic agents due to the critical role that plays among other cytokines in promoting joint inflammation and damage; the inhibition of TNFα has already contributed to successfully treat several patients. The current therapy with the combination of methotrexate and anti-TNF drugs has resulted in a marked amelioration of the disease severity and a significant reduction in radiographic damage [2]. Several biological agents targeting other cytokines or cytokine receptors or controlling immune cells including B and T lymphocytes are being used. Other molecules are under evaluation in clinical trials and are recommended for use in patients who fail to respond to TNF inhibitors [3], [4], [5].

There is a general consensus on the use of combination therapies to control the progression of the disease with the goal to neutralize the pro-inflammatory effect of different mediators [5]. Due to the multifactorial nature of the RA disease, the biological agents that are currently available for the management of RA patients are unable to control all the inflammatory mediators responsible for the onset, persistence and amplification of the inflammatory process in RA. The biologically active products of the complement (C) system are among the mediators that are not selectively neutralized by these agents. The finding, in several clinical studies, of C activation products in the synovial fluid and tissue of RA patients supports the contribution of C activated through the classical, alternative and lectin pathways to chronic synovitis in RA [6]. Further evidence was obtained from animals deficient in various complement components including C3 [7], factor B [7], properdin [8], C5 [7], C6 [9], MASP1/3 [10]. Similar results were observed in mice lacking C receptors [9], and regulators [11].

Based on these observations, inhibition of C activation is critically important in planning therapeutic strategies in order to prevent developing arthritis or to treat established joint inflammation [12]. C5 appears to be an ideal target to control because its activation leads to the release of the potent pro-inflammatory peptide C5a and to the assembly of the terminal C complex responsible for tissue damage and inflammation. Neutralizing antibodies to C5 [7], [13] or C5a receptor antagonist that interferes with C5a–CD88 interaction [14] have the advantage to control the attack phase of C activation leaving unimpaired the critical defence function that is mainly associated with the activation of C3.

A problem that has not yet been solved in the management of RA patients is how to reduce and possibly avoid the side effects, in particular the increased risk for common and opportunistic infections, that may be associated with the chronic administration of therapeutic drugs [2]. In addition, a treatment based on biologicals (such as monoclonal antibodies) for patients with chronic diseases such as RA requiring long term treatment is extremely expensive [15]. One innovative strategy for simultaneously lowering both the side effects and the cost is to deliver selectively the drug to inflamed synovium, as we recently demonstrated using the targeted recombinant antibody neutralizing C5 MT07 [16].

We now propose an alternative strategy based on the in vivo production of a neutralizing scFv-Fc fusion protein against human C5 after intraarticular injection of DNA vector.

Recombinant DNA technology has been used to improve plasmid *in vivo* protein production in order to overcome many of the problems associated with the use of natural allergen extracts, such as insufficient quality, allergenic activity, and poor immunogenicity. Numerous clinical trials have also demonstrated the many advantages of allergen-specific immunotherapy based on DNA injection over conventional pharmacotherapy [17].

Our aim is to use this technology in order to induce local production of the recombinant scFv-Fc anti-C5 miniantibody MB12/22 (Mubodina®, ADIENNE Pharma & Biotech, Italy) in sufficient amount to prevent complement activation in the joint and to prevent joint inflammation in experimental model of arthritis in rat.

## Materials and Methods

### Animals

Wistar rats were obtained from a colony kept in the animal house at the University of Trieste. Male

animals weighing 230–260 g were used in this study.

The experimental procedures were approved by the Italian Ministry of Health and by the Bioethic Committee of Trieste University in accordance with national (Legislative Decree 116/92 and law n. 413/1993) and international (Directive 86/609/EEC and the recommendation 2007/526/EC from European community) laws and policies. All treatments were performed under total anesthesia induced with 25 mg/kg bromoethanol (Avertin; Sigma, St. Louis, MO).

### C5 12/22 scFv cloning into pMB and PUCOE vector

Anti C5 antibody sequence [18] was cloned into pMB plasmid vector [19] containing rat Fc sequence. scFv-Fc fusion was then amplified and after restriction digestion subcloned into the pUCOE plasmid vector as described by Boscolo et al [20]. All cloning steps were check by DNA sequencing. As a control was cloned into pUCOE plasmid vector a DNA sequencing for an antibody unable to recognize murine structures.

### CHO-S transfections

Chinese Hamster Ovary subclone (CHO-S) cells were grown in CHO-S-SFM II plus penicillin (10 U/mL), streptomycin (1 µg/mL) and L-glutamine (2 mM) (all from Invitrogen) until transfections. Cells grown to confluence on 2 cm^2^ wells plate were transfected with FreeStyle™ MAX Reagent (Invitrogen) and 1 µg of selected expression vector, and culture supernatant was harvested 24–72 hours post-transfection for the analysis of antibody production. Growing conditions for cells were 5% CO2 in humidified atmosphere at 37°C.

### Enzyme-linked immunosorbent assays (ELISA)

The scFv-Fc secreted by pMB or pUCOE transfected-CHO-S cultures was assessed by ELISA. Multi well strips (Costar, Corning Incorporated) were coated with BSA, human C3 or human C5 at 0,5 mg/ml by overnight incubation at 4°C. After saturation with PBS containing 2% non-fat milk, the supernatant of CHO-S expressing scFv-Fc (diluted 1∶100) was added and incubated for 1 hour at 37°C. Bound scFv-Fc was detected by adding anti-SV5 mAb (1∶2000 in saturation buffer) [21] followed by HRP conjugated goat anti mouse Ig (Jackson Immunoresearch) (dilution 1∶1500 in saturation buffer). The enzymatic reaction was revealed using 3, 3′,5,5′-Tetramethylbenzidine Liquid Substrate (TMB) (Sigma-Aldrich) and the absorbance was read at 450 nm.

### Erythrocyte intermediates and hemolytic assays

Sheep red blood cells were sensitized with subagglutinating amount of rabbit IgM antibodies and resuspended in glucose veronal-buffered saline (GVBS). The lytic assay was performed by incubating 50 µl of antibody-sensitized erythrocytes (1.5×10^7^) in 150 µl of GVBS containing human or rat serum for 30 min at 37°C, and then reading the cell supernatant at 415 nm [18].

### Induction of AIA

The animals received 2 intradermal injections of an emulsion containing an equal volume of 100 µg of methylated bovine serum albumin (mBSA) in 200 µl of sterile saline and Freund's complete adjuvant (CFA) (both from Sigma-Aldrich), at the base of the tail. Fourteen days after the second injection, arthritis was induced by intraarticular administration of mBSA (100 µg in 100 µl of saline) into the right knee, while saline was injected into the left knee and served as a control. The development of arthritis was followed by measuring the swelling of the knees with a caliper. The animals were killed 3 days after the intraarticular administration of mBSA, the periarticular tissue of the knee joints was gently removed to expose the intraarticular cavity and lavage was performed with 2 ml of saline. Total cell count in the synovial lavage fluid was measured by ZB1 Coulter Counter (Beckman Coulter, Luton, UK,, and the number of polymorphonuclear neutrophils (PMNs) was evaluated measuring their myeloperoxidase content, as previously reported. Part of the anterior capsule of the joint was removed, embedded in OCT compound (Miles, Milan, Italy), snap frozen in liquid nitrogen, and kept at −80°C until used for immunofluorescence analysis. Knee joints fixed for 6 days in 10% buffered formalin, decalcified for 5 days in Decalcifier I (SurgiPath, Richmond IL), and embedded in paraffin were used for histologic analysis.

### Treatment of rats with DNA encoding MB12/22

Two experimental approaches were used to evaluate the biological activity of the anti-C5 scFv-Fc produced after DNA injection.

In the first set of experiments, healthy rats received an intraarticular injection of 0,1 ml of PBS containing liposomes (DMRI-C, 30 µg, Invitrogen) and DNA (6 µg) into the right knee of three rats. The animals were followed for 14 days. Efficacy of the transfection was evaluated by searching for the presence of DNA encoding scFv-Fc by PCR. Antibody production was detected by western blot using the SV5 specific antibody. Finally the toxicity of the treatment was evaluated by histological analysis of different organs.

In the second set of experiments the activity of locally synthesized scFv-Fc was evaluated injecting intra-articularly liposomes bearing the DNA prior to the intra-articular injection of mBSA. The time of injection and the concentration of liposomes were selected on the basis of the results obtained in the first set of experiments.

### Analysis of plasmid vector in synovial tissue

The efficacy of transfection was evaluated analyzing the presence of DNA encoding MB12/22 in the synovium obtained from different rats. Total DNA was extracted from lysed synovial tissue and used to amplify DNA that encodes the anti-C5 scFv-Fc employing the same primers used for its subcloning as reported by Boscolo et al. [20]. Amplification was detected separating DNA in agarose gel.

### SDS-PAGE and Western blot

Lavages of knee joints were subjected to SDS-PAGE on 10% gel under reducing conditions according to Laemmli followed by electrophoretic transfer onto nitrocellulose membrane (Hybond ECL; GE Healthcare, Milan, Italy) using the semidry Semiphor transfer unit (Heifer Scientific Instruments, San Francisco, CA). After soaking in 50 mM Tris-HCl pH 7.6 containing 0.5 M NaCl and 4% skimmed milk for 1 h at 37°C to block the free binding sites, the nitrocellulose sheet was incubated with 1/5000 alkaline phosphatase conjugated goat anti-rat IgG. The enzymatic reaction was developed using nitroblue tetrazolium (0.60 mg/ml) and 5-bromo-4-chloro-3-indolyl phosphate (0.30 mg/ml), both purchased from Sigma-Aldrich and diluted in 0.1 mM Tris-HCl pH 9.5 containing 0.1 M NaCl and 5 mM MgCl2. Rainbow RPN 756 (GE Healthcare) was used as a mixture of defined molecular markers.

### Immunofluorescence analysis

Tissue deposition of C3 was assessed by incubating frozen tissue sections with goat IgG anti-rat C3 (Cappel, ICN Biomedicals, Milan, Italy) at a 1∶200 dilution for 60 minutes at room temperature followed by FITC–labeled rabbit anti-goat IgG at a 1∶200 dilution (DAKO, Glostrup, Denmark) for additional 60 minutes at room temperature. A similar approach was used to examine synovial tissue for the presence of C9, using rabbit IgG anti-rat C9 (a kind gift from Prof. P. Morgan, Cardiff, UK) at a 1∶1000 dilution, followed by FITC labeled swine anti-rabbit IgG (DAKO, Glostrup, Denmark) at a 1∶40 dilution. The fluorescence intensity was analyzed in 10 different tissue areas (0,07 mm^2^ each) of each tisample using ImageJ software.

### Histomorphologic evaluation

Sections (6–8 µm) of paraffin-embedded synovial tissue obtained from animal models of arthritis were stained with hematoxylin and eosin and examined by two independent observers by using magnifications ranging from 25× to 400x. The degree of histomorphologic tissue damage was quantified by assessing a cumulative score resulting from the evaluation of different parameters related with the occurrence of the arthritic damage. Following a previously described method of analysis [22], the extent of joint inflammation was scored as follows: degree of synovial hyperplasia, from 0 to 2 (1–2 lines of regularly shaped synoviocytes = 0; 3–5 lines of irregularly shaped synoviocytes = 1; more than 5 lines of hypertrophied cells = 2), extent of leukocyte infiltration, from 0 to 3 (no infiltration = 0; scattered few infiltrating cells = 1; more diffuse infiltration, in a surface area <50% of the examined visible field = 2; a higher degree of tissue infiltration, or follicular aggregations = 3), presence of vascular lesions, from 0 to 2 (no lesions = 0; vasculitis or thrombosis in less than 50% of the visible/field vessels = 1; the same lesions in more than the 50% of the visible vessels = 2), percent diffusion of tissue fibrosis, from 0 to 3 (no alterations = 0; a 25–50% loss of the fat tissue component of synovial membranes = 1; more than 50% loss of the synovial fat tissue component = 2; complete derangement with 100% loss of synovial adipose components = 3). At least four specimens obtained from each treated knee joints were examined.

### Statistical analysis

The results were expressed as mean±SD. Data were compared by ANOVA using post-hoc analysis for paired multiple comparisons with Fisher's corrected t test. A nonparametric Mann-Whitney test was used to determine the significance of differences between tissue damage scores in the tested groups. (*) P values less than or equal to 0.02 were considered significant.

## Results

### In vitro characterization of DNA encoding for anti-C5 neutralizing antibody

**Figure 1:**
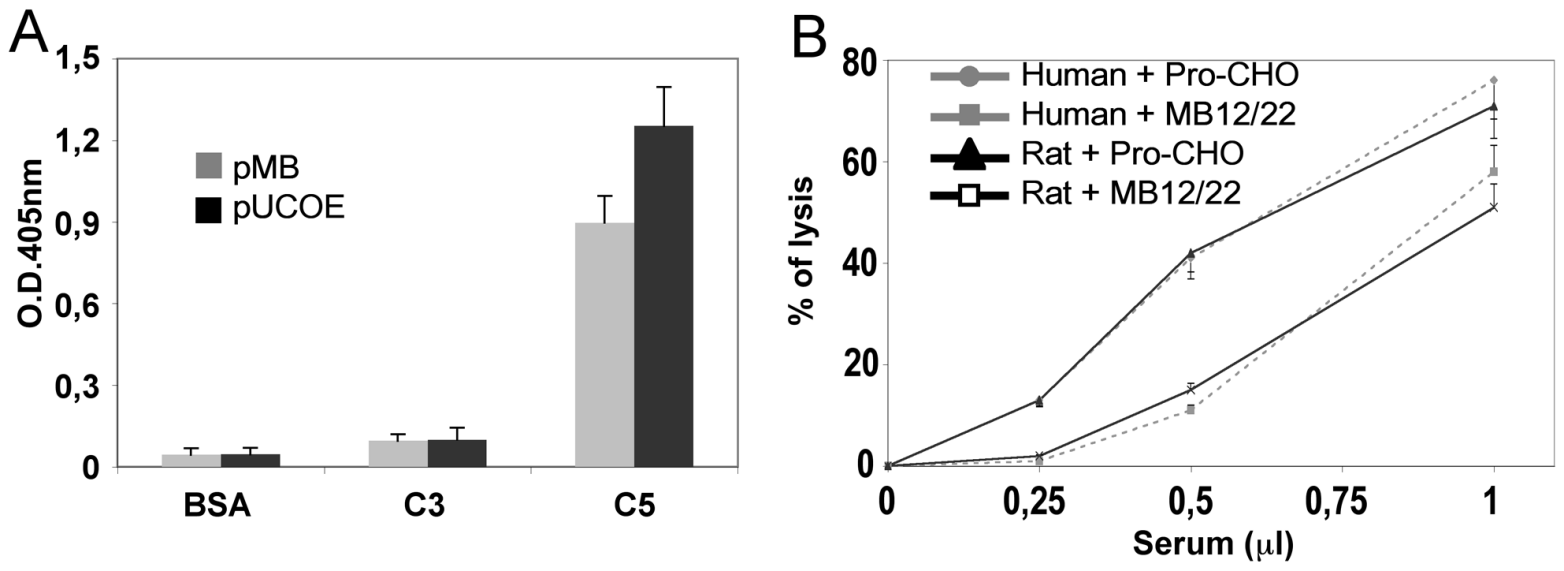
Effect of pUCOE vectors in the production of MB12/22 miniantibody. CHO-S cells were transfected with pUCOE or pMB plasmid vector containing MB12/22 encoding sequence. Miniantibody expression levels and specificity were monitored in the culture medium collected 72 hours after transfection analyzing the binding of the recombinant antibody to C5 but also on C3 and BSA by ELISA (A). Miniantibody activity was also controlled analyzing inhibition of human and rat C5 activity in the activation of the classical pathway of the complement system (B). Values are the mean ± SD.

We have efficiently produced scFv-Fc fusion proteins in the supernatant of transient and stable transfected HEK 293T or CHO-S cells [18], [22], [23], [24], [25]. More recently we have improve this strategy by using an optimized UCOE containing vector [20] that guarantees both a longer expression as well as an increased production yield of the recombinant protein of interest. In this study we selected the scFv TSA12/22 directed to C5 complement component. This scFv is able to neutralize the complement component C5 from man, and various animal species including rat, mouse and rabbit. The scFv was expressed as a scFv-Fc fusion protein containing the rat Fc region. In order to compare the performance of the standard pMB vector with the UCOE containing vector, CHO-S cells were transfected with both and the recombinant antibody released in the culture medium was tested for its ability to recognize C5. The results presented in Fig 1A confirm that both recombinant antibodies were able to bind human C5 as revealed by ELISA while failing to react with human C3 or BSA. As expected, pUCOE revealed a higher level of protein production and was used for the synthesis of MB12/22 for all subsequent experiments. The recombinant antibody maintained the ability to inhibit the C5 activity in a standard hemolytic assay of complement activation through the classical pathway and proved to be equally effective in blocking the activity of both human and rat C5 (Fig 1B).

### *In vivo* characterization of DNA encoding for anti-C5 neutralizing antibody

**Figure 2:**
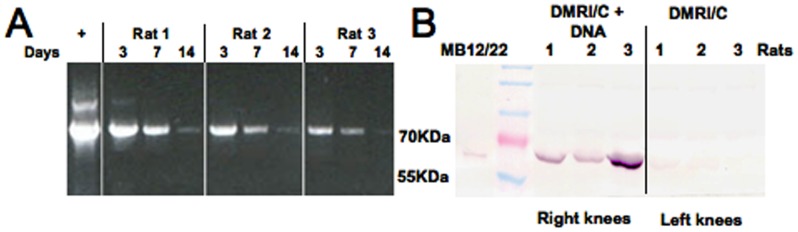
Effect of the intraarticular injection of DNA in the production of MB12/22. DMRI-C (30 µg)+MB12/22 DNA (6 µg) or DMRI-C alone were injected in the right knee of 3 animals per group. Miniantibody encoding DNA was detected in synovial tissues by PCR for 14 days (A). Miniantibody production was confirmed at day 3 post-injection in the washes of the right (but not of the left) knees by western blot (B).

*In vivo* DNA delivery and transfection was achieved by using DMRI-C reagent. The pUCOE vector encoding the recombinant molecule MB12/22 was purified and after appropriate reaction with DMRI-C was directly injected in the right knee of three groups of rats. As a control DMRI-C transfection reagent alone in PBS was administered to the left knee. The efficacy of Cationic lipid-mediated gene transfer was evaluated analyzing the synovial cells for the presence of DNA encoding MB12/22 by PCR at different time points after transfection. As shown in Figure 2A, DNA encoding the scFv-Fc was present in the synovial tissue. The peak was documented 3 days after DNA challenging in the right knee of all treated animals while it was absent in the controlateral knee and in other organs of the rats (data not shown).

The presence of the antibody in the synovial fluid was also evaluated at protein level by western blot. Fig 2B shows the band corresponding to the miniantibody MB12/22 detected at day 3 after DNA injection in the lavage obtained from the right knee of 3 rats. The band was hardly detectable at day 7 while undetectable at day 14 in the right knee as well as in the left knee throughout the whole period of observation (data not shown).

**Figure 3:**
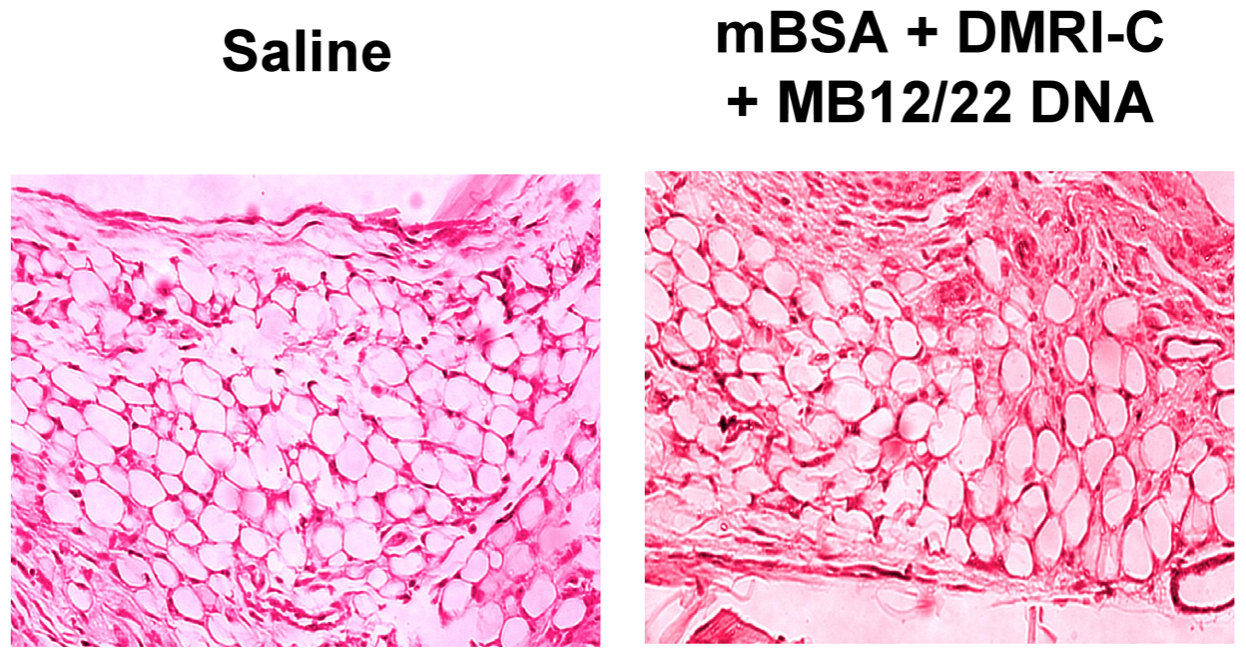
Effect of the intraarticular injection of DNA in the production of MB12/22. Rats were treated with either DMRI-C + MB12/22 DNA or saline as a negative control. Three days later, the animals were euthanized and the synovial tissue was analyzed. Note that the synovial tissue exhibit essentially a normal histologic structure in both groups of rats.

Histological analysis of synovial tissues revealed a normal structure and the absence of inflammatory cells, indicating the low toxic effect of DNA injection (Figure 3).

Rat sera were also collected and analyzed for complement activity that remained unchanged after treatment (data not shown).

### *In vivo* inhibition of complement activation

To evaluate the ability of MB12/22 to prevent complement-mediated tissue damage by inhibiting C5 cleavage, a model of antigen-induced arthritis (AIA) obtained by injecting (mBSA) in the rat knee was used. This experimental model was selected because the inflammatory process is restricted to a single joint enabling the comparison of the biochemical and structural changes with those of the unaffected controlateral joint. In addition, this model of monoarthritis is characterized by a ‘well defined’ time of onset, with the development of joint inflammation a few hours after intraarticular injection of mBSA [16], [18], [22]. Based on the finding that the production of MB12/22 reaches its peak 3 days after DNA administration (Figure 2), we decided to inject the DNA-conjugated liposomes 3 days prior to BSA injection. The aim was to control complement activation that has been recognized to be one of the initial events occurring in the acute phase of this disease and also responsible for amplification of the inflammatory process during progression of RA [7], [26], [27]. The animals were sacrificed 3 days after antigen challenge, when the maximum joints swelling, the highest number of PMNs in the articular wash fluids and the overt synovial damage is observed in the rat AIA model [18].

**Figure 4:**
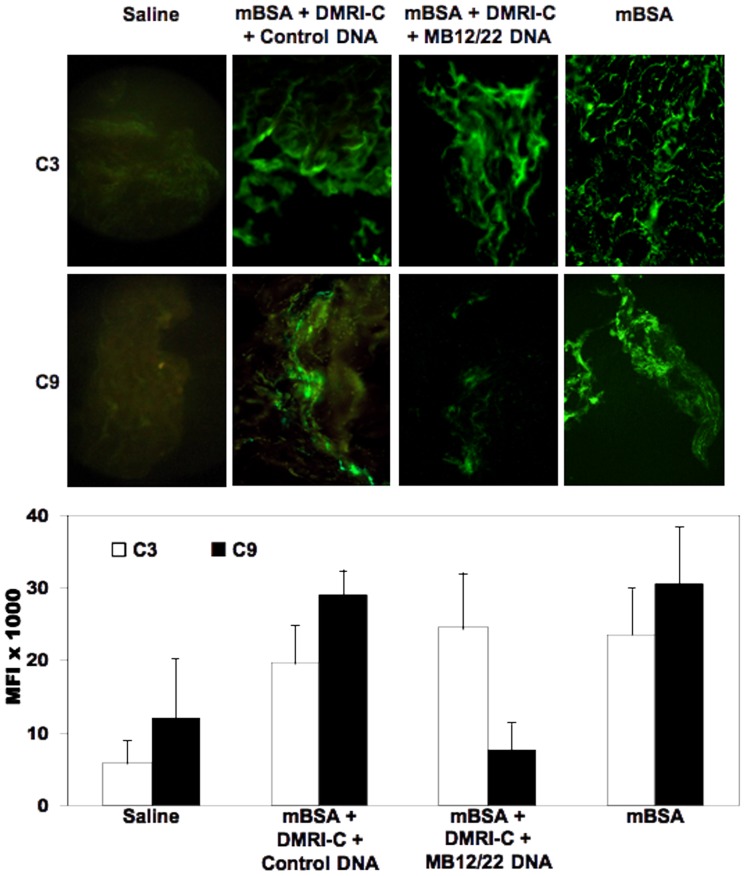
Effect of DNA encoding MB12/22 on the activation of the complement system in the model of antigen-induced arthritis in rats. Immunofluorescence analysis of synovial membranes for deposition of C3 and C9 obtained from rats receiving mBSA 3 days after intraarticular injection of Saline (indicated as BSA), DMRI-C + MB12/22 DNA or DMRI-C alone. The animals were sacrificed 3 days after arthritis induction and samples of synovial tissue were analyzed for deposition of C3 and C9. Saline-treated groups represent a negative control group in order to show the absence of complement activation. Note that while C3 is detected in the synovium of mBSA + saline, mBSA + DMRI-C containing control DNA animals, we failed to reveal binding of C9 on the synovial tissue of DMRI-C + MB12/22 DNA-treated rats. The graph in the lower part of the figure shows the mean fluorescence intensity (MFI) of the images ± SD. for each experimental group. (*): P values less than or equal to 0.02 were considered significant. Pictures were taken at original magnification 200X.

Analysis of synovial tissue removed from rats receiving either mBSA or mBSA plus liposomes bearing control DNA revealed marked deposition C3 and C9 along the synovial lining layer and in subsynovial connective tissue, whereas no staining was observed in the tissue harvested from saline treated rats used as a negative control (Figure 4). As expected, injection of liposomes bearing DNA encoding scFv-Fc MB12/22 into the rat knee treated with mBSA did not prevent binding of C3, which showed a distribution pattern and a staining intensity similar to that observed in rats treated with mBSA and empty liposomes. Conversely, a marked difference was seen in the deposition of C9, which was substantially reduced in the MB12/22 producing animals (Figure 4).

### Prevention of inflammation by MB12/22 DNA

**Figure 5:**
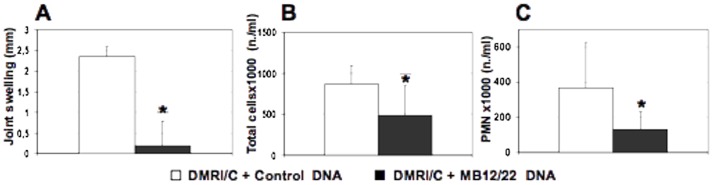
Effect of DNA encoding MB12/22 on the development of inflammation in the model of antigen-induced arthritis in rats. Rats were treated with mBSA 3 days after intraarticular injection of DMRI-C + MB12/22 DNA or DMRI-C alone. Three days later, animals were euthanized and examined for joint swelling (A) and total cells (B) or PMN (C) count in synovial lavage fluids. Values are the mean ± SD of 5 rats per group. (*): P values less than or equal to 0.02 were considered significant.

**Figure 6:**
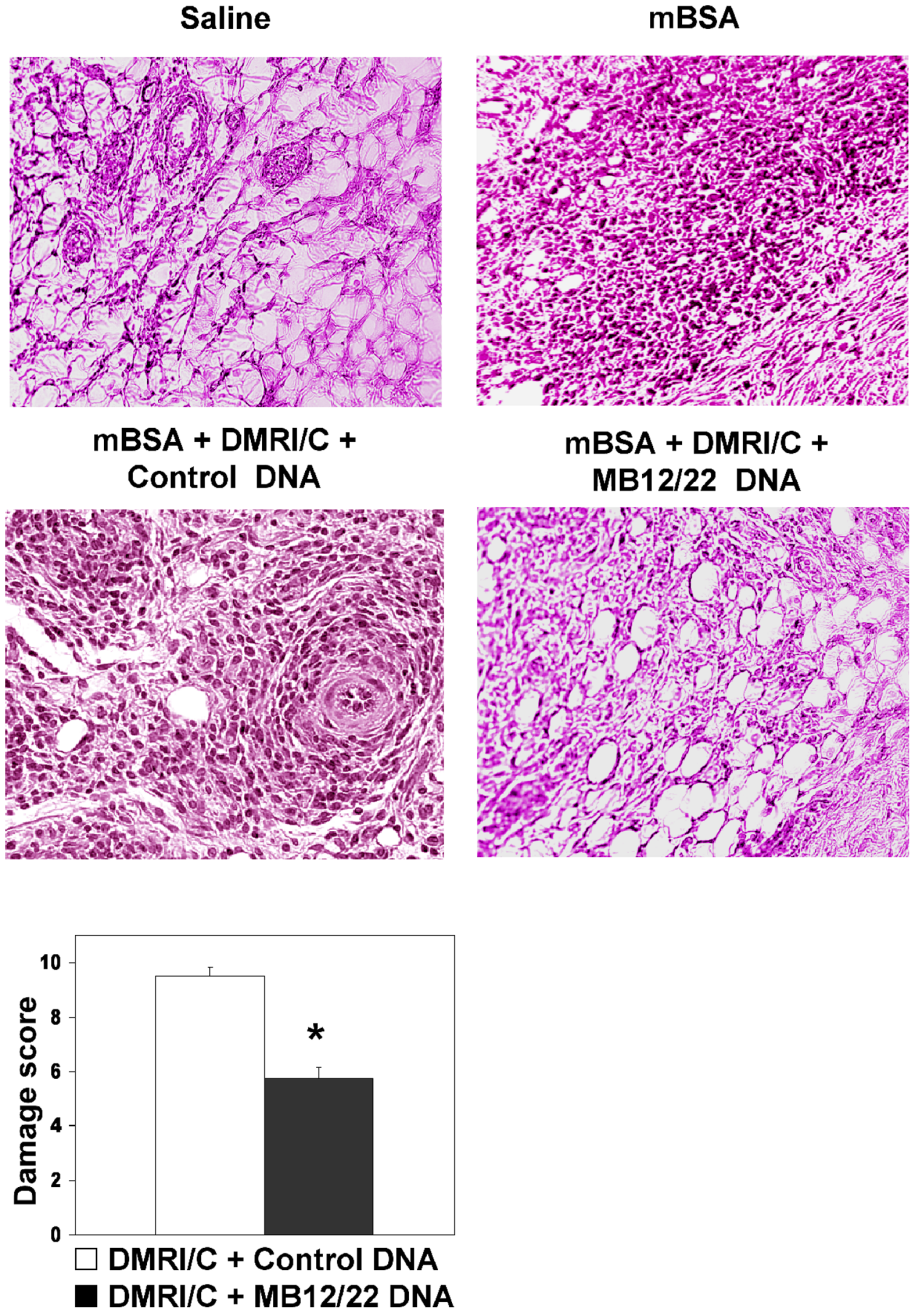
Effect of DNA encoding MB12/22 on the development of articular damages in the model of antigen-induced arthritis in rats. Rats were treated with mBSA 3 days after intraarticular injection of PBS, DMRI-C + MB12/22 DNA or DMRI-C + control DNA. Saline-treated groups represent a negative control group in order to show a normal synovia. Three days later, animals were euthanized and synovia tissues were analyzed. Note the synovial hyperplasia and leukocyte infiltration in the mBSA alone, mBSA + DMRI-C treated rats, as compared with the clearly milder synovial alterations of synovium in the DMRI-C + MB12/22 DNA rat. Original magnification 250×. A tissue damage score was determined as the degree of synovial hyperplasia, cell infiltration, vascular lesions, and tissue fibrosis. Values are the mean ± SD of 5 rats per group. (*): P values less than or equal to 0.02 were considered significant.

Liposomes containing anti-C5 DNA administered to rats undergoing AIA caused a marked reduction in joint swelling (Figure 5A) and a significant decrease in total cell (Figure 5B) and PMN number (Figure 5C) as compared to empty liposomes. Histological analysis of the joints obtained from rats receiving mBSA or the mixture of BSA and liposomes conatining control DNA revealed the presence of hyperplasia of lining synovial cells and marked leukocyte infiltration in the synovial tissue. Conversely, synovial hyperplasia, leukocyte infiltration, vasculitis and fibrosis were significantly reduced in rats after a single treatment with anti-C5 DNA (Figure 6). The difference in the histologic changes observed in the various groups of rats was also confirmed by the finding that damage score obtained by a quantitative evaluation of synovial hyperplasia, leukocyte infiltration, vasculitis and fibrosis was significantly reduced in animals treated with liposomes containing MB12/22 vector compared to control DNA (Figure 6).

## Discussion

Delivery of anti-inflammatory drugs to inflamed tissue represents an ideal therapeutic strategy to control inflammatory process in chronic diseases including rheumatoid arthritis. This approach allows a selective accumulation of the drug at tissue level thus avoiding side effect that may derive from long-term systemic administration of therapeutic drugs. The risk of tuberculosis reactivation and opportunistic bacterial infection is a pitfall of treatment with anti-TNF and other biologic agents [2].

We have previously reported the characterization of the recombinant antibody MB12/22 originally selected for the ability to recognize human C5 that the unique property to cross-reacts with C5 from several species, including mice, rats and rabbits [18], and thus allows *in vivo* validation of its biological effect in preclinical models before its therapeutic use in patients. We have shown that two antibodies to C5 injected intra-articularly are able to reduce joint swelling, cell counts and tumor necrosis factor levels in synovial lavage fluids, and histomorphologic changes [18], [22].

We have now taken the approach of local administration of MB12/22 one step further, injecting DNA encoding the antibody directly into the knee joint. A single intraarticular injection of a plasmid vector encoding MB12/22 in complex with DMRI-C was sufficient to induce production of the recombinant molecule in only 3 days and the presence of MB12/22 DNA in synovium was detected up to 14 days after challenging. DMRI-C is a liposomal formulation that was selected for the ability to transfect efficiently mammalian cells following interaction with nucleic acids. Cationic liposome-mediated *in vivo* gene transfer was already performed using DMRI-C in mouse, rat and pig with a very low toxicological profile [28].

DNA transfections have several conceptual advantages over traditional drug therapy. DNA plasmids are relatively simple and inexpensive to design and create, and are also easier to purify. The high stability and the relative temperature insensitivity make these reagents highly suitable for mass production and distribution [29]. DNA technology has already been employed for the preparation of vaccines, overcoming many of the problems associated with the use of natural allergen extracts, such as insufficient quality, allergenic activity, and poor immunogenicity. Numerous clinical trials have also demonstrated the many advantages of allergen-specific immunotherapy over conventional pharmacotherapy [17].

Viral transfection is used as another strategy for *in vivo* gene transfer with the aim to induce production of proteins including recombinant antibodies. However, potential insertion of viral DNA into the cell genome and formation of antibodies to viral proteins, which may reduce viral persistence and limit the transfection efficiency, represent two major drawbacks of this technical approach [19], [30], [31].

Recombinant DNA technology enables the *in vivo* production of large amount of antibodies with complete human sequence, conserving the same glycosylation pattern of native immunoglobulins, and are therefore less immunogenic after repeated administration to patients requiring long-term treatment [32]. In addition, these antibodies are available at relatively low cost and their cDNA can be delivered to tissue sites by non-invasive means [33].

Local production of neutralizing anti-C5 recombinant antibody after a single DNA injection was sufficient to induce an anti-inflammatory effect. Our results show that this preventive approach was as efficient as the intraarticular injection of the anti-C5 recombinant antibody MB12/22 in reducing inflammation in a rat model of antigen-induced arthritis [18], [22]. This conclusion is supported by the a lower increase in swelling and the results of the histological analysis, showing reduction in synovial hyperplasia, leukocyte infiltration, particular of PMN, and vascular lesions.

AIA induced in rats represents a good model of monoarthritis and its onset and maintenance is mainly due to local activation of the complement system [34], [35]. Complement involvement in AIA is confirmed in the present study by the observation of marked deposition of C3 and C9 in the synovial tissue of immunized animal receiving booster intrarticular injection of BSA. The finding of reduced deposits of C9 in rats that had received intraarticularly plasmid vector encoding MB12/22 prior to BSA injection is a clear indication that the locally produced antibody was able to prevent to a large extent complement activation. As expected, the neutralizing effect of MB12/22 directed against C5 was restricted to the terminal pathway and did not affect C3 deposition. The milder manifestation of arthritis observed in rats treated with the plasmid vector confirm our previous observation that the activation products of the late complement components including C5a and C5b-9 are mainly responsible for the inflammatory process developing in the knee joints in rats undergoing AIA.

Overall these findings support the beneficial effect of local neutralization of complement activation to control joint inflammation. We believe that the intrarticular injection of plasmid vector encoding recombinant antibodies may be adopted as a novel preventive approach to treat monoarthritis as an alternative to local treatment with antibodies commonly used in this form of arthritis [36], [37] with the advantages of the lower cost and the longer persistence of antibody production.
